# Microbial Regulation of Glucose Metabolism and Insulin Resistance

**DOI:** 10.3390/genes9010010

**Published:** 2017-12-29

**Authors:** Silke Crommen, Marie-Christine Simon

**Affiliations:** 1 Department of Nutrition and Food Sciences, Nutritional Physiology, University of Bonn, 53115 Bonn, Germany; S.crommen@uni-bonn.de; 2 The Wallenberg Laboratory, Department of Molecular and Clinical Medicine, University of Gothenburg, 41345 Gothenburg, Sweden

**Keywords:** diabetes, insulin sensitivity, diet, gut molecules, microbiota, probiotics

## Abstract

Type 2 diabetes is a combined disease, resulting from a hyperglycemia and peripheral and hepatic insulin resistance. Recent data suggest that the gut microbiota is involved in diabetes development, altering metabolic processes including glucose and fatty acid metabolism. Thus, type 2 diabetes patients show a microbial dysbiosis, with reduced butyrate-producing bacteria and elevated potential pathogens compared to metabolically healthy individuals. Furthermore, probiotics are a known tool to modulate the microbiota, having a therapeutic potential. Current literature will be discussed to elucidate the complex interaction of gut microbiota, intestinal permeability and inflammation leading to peripheral and hepatic insulin resistance. Therefore, this review aims to generate a deeper understanding of the underlying mechanism of potential microbial strains, which can be used as probiotics.

## 1. Introduction

The increasing prevalence of obesity, combined with changing dietary habits and exercise, seems to reach epidemic proportions worldwide. As more than 80% of patients with type 2 diabetes (T2D) are overweight, obesity appears to be a significant factor in the increasing incidence of T2D in the world [1,2]. Also, the proportional increase in T2D shows this alarming trend. From 1980 to 2008 the number of people diagnosed with diabetes, of which 90% are patients with T2D, increased from 153 million to 347 million [3,4]. Given the substantial health economic consequences of obesity and diabetes, further research to better understand the pathophysiological processes and to develop new therapeutic approaches is needed.

## 2. Insulin Resistance in the Development of T2D

T2D results from decreased insulin sensitivity in combination with insufficient insulin secretion. When approximately 65% of the β-cell function is lost and occurring insulin-resistance cannot be compensated by hyperinsulinemia, T2D becomes overt [5,6,7,8,9,10]. Moreover, T2D is associated with reduced incretin concentrations as well as incretin effect [11,12,13,14], resulting in an impaired insulin secretion in response to glucose. In particular, the first phase of insulin secretion is diminished in T2D, indicating the important role of incretins in diabetes development [15]. 

**Figure 1:**
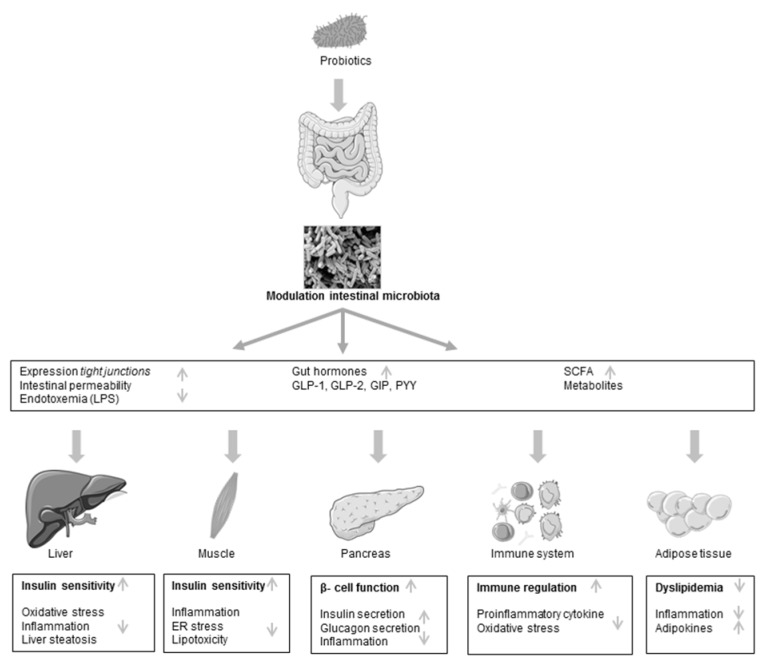
Schematic view of postulated mechanism of probiotic action in type 2 diabetes. LPS, lipopolysaccharides; GLP, glucagon-like peptide; GIP, gastric inhibitory polypeptide; PYY, peptide YY; SCFA, short-chain fatty acid; ER, endoplasmatic reticulum.

Insulin resistance, described as the fundamental failure to respond appropriately to insulin, mainly affects the target tissues of insulin, particularly skeletal muscle and liver, but also adipose tissue and brain [9,16,17,18,19,20,21,22] (Figure 1). Whether the peripheral or the hepatic insulin resistance occurs first and what is the driving feature is still under debate. Skeletal muscle insulin resistance, in terms of dysfunction of cellular mechanisms to respond appropriately to insulin, and the resulting reduction of peripheral glucose uptake seem to develop early, as shown by studies in young lean individuals with muscle-specific insulin resistance [23]. As a consequence, glucose is redirected to the liver, which increases de-novo-lipogenesis with consecutive impairment of hepatic energy metabolism [24,25,26]. On the other hand, it has been suggested that hepatic insulin resistance is the primary event initiating the development of diabetes. Thus, disruption of hepatic insulin signaling results in fasting and postprandial hyperglycemia and the subsequent development of peripheral insulin resistance [27,28]. 

The link between elevated lipid levels and insulin resistance is widely accepted. Increased availability of free fatty acids (FFA) and subsequent ectopic intracellular lipid accumulation may trigger the development of insulin resistance. Particularly, an increased intracellular lipid content in skeletal muscle and liver has been related to insulin resistance [29,30]. It was postulated that, in muscle and liver, the intracellular accumulation of lipids and diacylglycerol (DAG) triggers the activation of novel protein kinases Cs (PKCs) with subsequent impairment of insulin signaling. For example, insulin-receptor substrate (IRS) 1-associated phosphatidylinositol 3-kinase (PI3K) activity is reduced in the muscles of individuals after a lipid infusion. In addition, in these individuals the insulin action in the liver, which has some similarities with the insulin action in muscle, is associated with defects in insulin signaling, e.g., PKCε activation, reductions in insulin-stimulated insulin receptor substrate-2 (IRS-2) tyrosine phosphorylation, in the state of hepatic steatosis. Increased liver lipid content further impairs the ability of insulin to regulate gluconeogenesis and activate glycogen synthesis [24,31].

Several other aspects, including genetic factors, have been described to contribute to alterations of insulin resistance [32,33,34,35,36,37,38,39,40]. It has been demonstrated that first-degree relatives of type 2 diabetic subjects have a higher risk to develop insulin resistance and subsequent type 2 diabetes [41,42,43,44]. However, the recent increase in the global incidence of T2D, which is observed in Western countries and developing nations, suggests that most cases of this disease are caused by changes in environmental factors. Major risk factors for T2D such as overnutrition and low dietary fiber involve the gut and have been found to be associated with increased insulin resistance, decreased glucose tolerance and local or systemic low-grade inflammation [45].

## 3. The Impact of the Intestinal Microbiota on T2D

The human intestinal tract is colonized with a plurality of microorganisms, consisting of numerous bacteria, archaea and viruses. This microbiota results in a biomass of about 1.5 kg. The microbiome, the number of genes of all bacteria localized in the intestinal tract, exceeds the human genome at least 500-fold [46,47,48]. Previous research mainly focused on the beneficial functions of these bacteria, such as the digestion of complex carbohydrates or the development of innate and acquired immunity. These studies also provide evidence that the microbiota may influence important functions in the regulation of metabolism in health and disease [49,50,51]. 

The human organism lives in a mutual symbiosis with the bacteria in the gut, and the development of culture-independent sequencing technologies with high-throughput metagenomic sequencing in recent years allows a better understanding of the complexity of the intestinal microbiota [52]. 

In a healthy symbiosis, the gut microbiota promotes development and maturation of the immune system and contributes to metabolic processes, such as the production of vitamins and the digestion of dietary fiber [53]. Since germ-free (GF) mice allow to investigate the mechanism behind this mutual symbiosis, new experimental options have arisen. In these studies, it has been observed that GF mice are leaner and gain less weight under a high fat diet, compared to their conventionally colonized littermates, despite a higher total food intake [54]. Obese mice showed a lower proportion of the dominant bacteria Bacteroidetes and a higher proportion of Firmicutes, compared to their lean littermates [55,56,57]. Later, these differences could also be shown between obese and lean patients [57,58]. In addition, weight reduction resulted in an increase of relative abundance of Bacteroidetes, up to 20%, and decreased abundance of Firmicutes, of about 10%, similar to the ratio of Bacteroidetes to Firmicutes of lean participants. These results indicate that obesity is influenced by the diet itself, but also by the gut microbiota, and this may provide individualized therapeutic opportunities [57]. However, while the ratio of Bacteroidetes and Firmicutes or their individual relative abundance seems to be associated with obesity in animal studies and several human studies, a recent meta-analysis revealed that this association was relatively weak and its detection might be confounded by large interpersonal variation and insufficient sample sizes [59]. 

The transfer of obese animals’ microbiota to GF mice resulted in higher body weight gain compared to these GF animals receiving microbiota from lean animals [56] The transfer of the microbiota to GF mice increased the body fat of these animals within two weeks by 60%, in spite of reduced food intake [60]. These results lead to the assumption that the obese microbiota is more efficient at yielding energy from the diet. Germ-free mice are a valuable tool for studying the ecosystem and metabolism of the human and animal intestinal microbiota, but it has some limitations. Importantly, the gut barrier permeability is markedly altered in germ-free mice, which makes their intestine permeable to inflammatory lipopolysaccharides. This might explain their proneness to alterations in energy homeostasis or metabolic control when they are colonized with microbiota [61]. Thus, the impact of gut microbiota dysbiosis on host metabolism by describing the beneficial effects of the transfer of dysbiotic gut microbiota has to be taken into consideration. 

In view of the promising results of fecal microbiota transplantation (FMT) in animal models, a double-blind, randomized, clinical trial investigated the effect of FMT in obese individuals with metabolic syndrome, showing an improvement of insulin sensitivity in those participants who received FMT from a lean donor, whereas the control group who received their own microbiota remained at stable insulin sensitivity. Also, the hepatic insulin sensitivity tended to be improved in the intervention group. The effect of FMT was accompanied by an increase in microbial diversity in the gut [62]. However, FMT still remains to be controversial and not all lean microbiota donors had beneficial effects on their obese recipient. The reasons for this, as well as possible side effects, should be further investigated. So far, the transfer of fecal microbiota has been clinically used for treatment of *Clostridium difficile* infections [63], suggesting the therapeutic potential of the microbiota [64].

Furthermore, Vrieze et al. [62] reported an association of insulin resistance with altered microbial diversity. Also, metagenomics studies showed that patients with T2D suffer from microbial dysbiosis, with a reduced abundance of butyrate-producing bacteria and an increase of opportunistic pathogens such as *Bacteroides caccae*, *Clostridia* and *Escherichia coli* compared to healthy persons. Both Karlsson et al. [65] and Qin et al. [66] independently reported a decreased number of butyrate-producing bacteria such as *Roseburia* and *Faecalibacterium prauznitzii* in the microbiota of patients with T2D compared with healthy subjects. In the study of Qin et al., the gut microbiota of 345 Chinese participants were examined. The authors reported a difference of 3% of the microbial genes and interpret this as a moderate dysbiosis in patients with T2D [66]. This cautious interpretation may be due to the limitations of the study, since there was no balanced age and gender distribution in the groups and information on current medication of the patients was missing. In the study of Karlsson et al., 145 postmenopausal Scandinavian women with normal glucose metabolism, impaired glucose tolerance or T2D were examined. Increases in the abundance of *Lactobacillus grasseri*, *Streptococcus mutans* and *Escherichia coli* was reported to be predictive of the development of insulin resistance in postmenopausal obese females in Sweden [65]. In order to develop a valid predictive marker based on the microbial composition, for example in obese individuals with increased risk of developing T2D, further investigations of the microbiota in clinical studies are needed [66,67,68,69,70]. 

Moreover, Wu et al. [71] showed that metformin, as firstline treatment for T2D, but not calorie-restriction had a strong impact on the microbial composition in individuals with newly-diagnosed T2D. Changes in composition of the microbiota, induced by metformin, were accompanied by an improvement of HbA1c and fasting blood glucose concentrations and even transferable to mice after colonization with microbiota of metformin-treated donors. Therefore, the authors stated that the anti-diabetic effect of metformin is due to an altered composition of the microbiota. 

Thus, it needs to be established whether the changes in the composition of the microbiota in T2D is a secondary effect, as a consequence of an altered intestinal motility, diet, drug therapy or bacterial overgrowth of the small intestine, as it is frequently observed in patients with T2D.

## 4. Diets and the Metabolic Products of the Intestinal Microbiota on Diabetes Development

Dietary habits of the Western lifestyle, such as consumption of fast food, are associated with insulin resistance [72]. In addition, high-fat diet [73,74,75] and reduced consumption of dietary fiber, especially cereals and/or carbohydrates with low glycemic index, are associated with insulin resistance [76,77,78,79,80,81,82,83,84]. It has been suspected that the consumption of dietary fiber is beneficial in several aspects. For instance, it can increase the production of short-chain fatty acids (SCFA) in the colon [85,86], which in turn may improve lipid homeostasis and reduce hepatic glucose output [87]. These metabolic alterations are mediated by the secretion of gastrointestinal hormones like ghrelin, peptide YY (PYY), and glucose-dependent insulinotropic peptide (GIP), with subsequent alteration of satiety [82,88,89,90,91,92]. Hence, the mechanisms through which these different diets promote the progression to insulin resistance and consecutively towards a pre-diabetic state involve a complex physiology of glucose homeostasis [93] and microbial metabolism [94], requiring further research. Additionally, it should be noted that the concept that different dietary components can modulate the microbiota may also be used therapeutically.

Also, the total energy intake and the macronutrient composition of the diet have an impact on the composition of the human gut microbiota and thereby on human health [95]. However, the gut microbiota respond rapidly to dietary interventions, since short-term consumption of diets, with either animal or plant products, can alter the overall community structure of the gut microbiota. Thus, an animal-based diet seems to increase the abundance of bile-tolerant microorganisms (*Alistipes*, *Bilophila* and *Bacteroides*) and decrease the levels of Firmicutes that metabolize dietary plant polysaccharides (*Roseburia*, *Eubacterium rectale* and *Ruminococcus bromii*) [96,97,98]. But in the long term, individual dietary preferences seem to affect the microbial community structure of the microbiota [98]. 

Furthermore, Wu et al. [98] showed that the gut microbiota of people consuming high amounts of protein and animal fat is dominated by the Bacteroides genus while the gut microbiota of people consuming more fiber and carbohydrates is dominated by the Prevotella genus. Similar findings were observed in a study comparing children in Burkina Faso and Italy, demonstrating reduced levels of Prevotella and increased levels of Bacteroides in the Italian cohort. The reduction in Bacteroides correlates with lower fiber intake [99]. Kovatcheva-Datchary et al. compared the composition of gut microbiota of healthy subjects who showed enhanced glucose metabolism after three-day consumption of barley kernel-bread (BKB) with subjects who did not respond (non-responder) to the dietary intervention. The Prevotella/Bacteroides ratio was higher in the responders than in non-responders after BKB consumption. Furthermore, metagenomics analysis revealed that microbiota was enriched in *Prevotella copri* and this was accompanied by an increase in the potential to ferment complex carbohydrates after BKB. Results of GF mice models transplanted with microbiota from responder human donors suggest that improvement in glucose metabolism might be promoted by increased glycogen storage [100].

However, clinical studies comparing omnivores and vegans indicate that the microbial metabolism in omnivores and vegans differs, since they reveal distinct metabolic profiles in the plasma, while there was no clear taxonomic shift in the microbial composition, which suggests that the microbiota adapts to shifts in diet which facilitate digestion of specific nutrients [101,102]. A study by Sonnenburg et al. [103] showed that changes in the microbiota of mice consuming a diet low in dietary fiber and harboring a human microbiota are reversible within a single generation, but not over several generations, where a diet low in fiber results in a progressive loss of diversity which is not recoverable after the reintroduction of dietary fibers. 

## 5. Probiotics

Probiotics are live microorganisms which, when ingested in adequate amounts, may exert specific health benefits to their host [104]. Since it is well accepted that disturbance in the intestinal microbiota is involved in the development of metabolic diseases, modifying the microbiota by probiotics seems to be a potential strategy in the prevention and treatment of diabetes. The anti-diabetic effects of probiotics have been extensively covered and demonstrated in animal studies [105,106,107,108,109,110,111]. Recently, Li et al. [109] investigated the anti-diabetic effects of *L. casei* CCFM419 in mice with high-fat diet and low dose streptozotocin-induced T2D. After four weeks, the probiotic group showed improved oral glucose tolerance, already at 30 min, and the area under the glucose response curve (AUC_Glucose_) was decreased compared to the diabetic control (DC) group. Also, after 12 weeks both the positive control group, treated with pioglitazone, and the probiotic group had significantly reduced AUC_Glucose_ values (27% and 25%, respectively) compared to the DC (*p* < 0.05). 

Furthermore, supplementation of *L. casei* CCFM419 ameliorated insulin sensitivity by insulin tolerance test, reduced fasting insulin level and decreased HOMA-IR value compared to the DC. The authors stated that *L. casei* CCFM419 contribute to an improvement in glycemic control over a long period of time as indicated by lowered HbA1c. In addition to the described effects, administration of *L. casei* CCFM419 was accompanied by reduced low-density lipoprotein cholesterol (LDL-C) level and increased high-density lipoprotein cholesterol (HDL-C) level. The positive effects of *L. casei* CCFM419 on hyperglycemia and insulin resistance may be due to improvement of STZ-induced damage of islet cells, PI3K/Akt signaling pathway, amelioration of systemic inflammation as indicated by improved TNF-α, IL-6 and IL-10 level and SCFA/gut microbiota pathways. Thus, these results suggest that oral administration of *L. casei* CCFM419 could delay the onset of hyperglycemia and improve impaired glucose tolerance [109]. Eventually, *L. lactis* strain genetically modified to produce GLP-1 is capable of improving glucose tolerance and stimulating insulin secretion in mice [112]. 

The efficiency of probiotics on diabetes has been linked to local changes of the gut environment and microbiota, reduction of the intestinal permeability and preventing translocation of bacterial lipopolysaccharides (LPS) in the systemic circulation [113] as well as stimulation of secretion of SCFA such as butyric acid in the colon and increased incretin secretion [109] (Figure 1). Furthermore, anti-oxidative, anti-inflammatory and immunomodulatory effects [106,108,110] are reported to be involved in the regulatory mechanism, preventing diabetes progression (Figure 1). According to this, in a study by Park et al. it was shown that administration of *L. rhamnosus* GG reduced infiltration and activation of macrophages in the adipose tissue. Additionally, endoplasmatic reticulum stress in skeletal muscle and lipotoxity, which is an important contributor leading to insulin resistance, was alleviated in *L. rhamnosus* GG-treated mice [108]. 

In contrast to the rather clear outcome of animal studies, there is controversial evidence from clinical studies concerning the effect of probiotic supplementation and T2D. An overview of studies which investigate the effect of probiotic supplementation in clinical trials in T2D patients is presented in Table 1. In several different studies, it has been demonstrated that administration of probiotics reduces insulin resistance, fasting blood glucose and HbA1c level [114,115,116,117,118,119].

A recent study conducted by Firouzi et al. [119] investigated the effect of multistrain probiotic supplementation on glycemic control, inflammatory marker, lipid profile and blood pressure in 136 individuals with T2D and found that supplementation of *Lactobacillus acidophilus*, *L. casei*, *L. lactis*, *Bifidobacterium bifidum*, *B. longum* and *B. infantis* (3 × 10^10^ cfu/g) for 12 weeks resulted in a modest reduction in HbA1c levels in comparison to placebo. Furthermore, insulin levels decreased in the probiotic group whereas insulin levels in the control group increased. The application of probiotics had no effects on inflammatory markers or on the lipid profile and blood pressure. Similarly, in another study by Ostadrahimi et al. [117] the administration of probiotic fermented milk containing *L. casei*, *L. acidophilus* and *B. lactis* for 8 weeks was found to reduce HbA1c in diabetic patients. 

*B. animalis subsp. lactis Bb12* and *L. acidophilus La5* have been shown to improve glycemic control in T2D patients [115,116]. Mohamadshahi et al. [116] reported a significant decrease in HbA1c after ingestion of probiotic yogurt for 8 weeks. The reduction in HbA1c was accompanied by decreased levels of proinflammatory cytokine TNF-α. Similarly, in a study by Ejtahed et al. [115], the ingestion of probiotic yogurt containing *B. animalis subsp. lactis Bb12* and *L. acidophilus La5* for 6 weeks reduced fasting blood glucose (8.68%, *p* < 0.001) and HbA1c, and increased the erythrocyte superoxide dismutase and glutathione peroxidase activities, as well as total anti-oxidative status compared with the control group who received conventional yoghurt. Additionally, the probiotic yogurt decreased total cholesterol (4.45%), LDL-C (7.45%) and the total-cholesterol:HDL-C and LDL-C:HDL-C as atherogenic indices in the intervention group [120]. Tonucci et al. [118] investigated the impact of administration of *B. animalis subsp. lactis Bb12* and *L. acidophilus La5* as probiotic fermented goat milk vs. conventionally fermented goat milk on glycemic control, lipid profile, marker of oxidative stress, cytokine level and fecal SCFA. Although the ingestion of fermented milk significantly reduced TNF-α and resistin level and increased acetic acid in fecal samples in both the probiotic and the control group, only the probiotic intervention group showed an improvement in glycemic control as indicated by reduced fructosamin levels and a trend in decreased HbA1c. The aforesaid findings indicate that *B. animalis subsp. lactis Bb12* and *L. acidophilus La5* could ameliorate glycemic control and improve some risk factors such as oxidative stress and dyslipidemia in diabetic patients. 

In contrast to these findings, Ivey et al. [121] observed no effects on glycemic control parameters in overweight participants by using the same probiotic strains applied as a yogurt alone or in combination with a probiotic capsule (containing additional 3 × 10^9^ cfu) for 6 weeks. Moreover, administration of *L. reuteri* induced increased GLP-1 and insulin secretion without altering glucose tolerance and insulin sensitivity in metabolically healthy overweight participants [122]. In another study, Mobini et al. [123] investigated the impact of supplementation with *L. reuteri* DSM 17938 in high (10^10^) or low (10^8^) dosage for 12 weeks in diabetic patients. It was shown that supplementation with *L. reuteri* DSM 17938 had no effect on HbA1c, liver steatosis, adiposity or microbiota composition. Even type 2 diabetes patients receiving the highest dose, presented only a trend to an increased insulin sensitivity-index (ISI) and a rise in secondary bile acid (deoxycholin acid). Additionally, it was shown that individuals who respond with improved ISI exhibited higher microbial diversity at baseline. 

However, the inconsistency in results obtained from clinical trials might be attributed to heterogeneity of the studied collective, including ethnicity, metabolic state, treatment and diabetes duration as well as intervention period and probiotic strains. Thus, there is large inter-individual variation in the response to dietary intervention in general and probiotic supplementation in particular. Reported studies support the hypothesis that the baseline microbial composition is of relevance for the successful stratification of patients and should be used to identify subjects who will respond to dietary intervention such as probiotic supplementation [100,123,124,125]. This should be taken into consideration in future studies. 

## 6. Genetic Background Affects Microbiota

Several other aspects, including genetic factors, have been described to contribute to alterations of insulin resistance [32,33,34,35,36,37,38,39,40,128]. It has been demonstrated that first-degree relatives of type 2 diabetic subjects have a higher risk to develop insulin resistance and subsequent type 2 diabetes [41,42,43,44]. 

Arising from a complex set of interactions between genetic risk factors and environmental influences, obesity, metabolic syndrome and T2D present as a spectrum of overlapping phenotypes from metabolically healthy obese individuals to those with full-blown T2D. In both humans [129,130] and rodents [131,132,133], genome-wide association studies have identified multiple loci that may contribute to obesity and its associated metabolic abnormalities, each with a small effect [134]. 

Furthermore, the microbiota contributes to metabolic disorders as indicated in recent studies in both rodents and humans [49,135]. Obese humans and rodents have less diverse gut communities than their lean counterparts [58,136,137], and likewise, metagenomic studies have documented differences in the microbiota represented in the gut communities of individuals with obesity and T2D [65,138,139]. Additionally, evidence for a causal relationship between the gut microbiota and metabolic dysfunctions has been shown by co-housing studies [140,141] and antibiotic treatment experiments [142,143] which can modify obesity and metabolic phenotype in rodents. 

Transplantation of fecal microbiota from obese versus lean mice, obese versus lean humans, and human twin pairs stably discordant for obesity into germ-free mouse recipients transmits donor adiposity and metabolic phenotypes [144,145,146]. However, much less is known about the relationship between microbiota and type 2 diabetes. Additionally, in permissive genetic backgrounds, environmental reprogramming of microbiota can ameliorate development of metabolic syndrome, as shown by conducting longitudinal analyses of the responses of three commonly used inbred strains of mice to long-term environmental conditioning as well as to shorter-term dietary challenges. Thus, there is a strong relationship between specific metabolic phenotypes and specific bacterial communities, indicating the strong, complex and dynamic interactions between the microbiota, diet, environment and host genetics [134].

## 7. Conclusions

The discovery of the differences in the composition of the microbiota in obesity and T2D was a crucial step in this research field. While cross-sectional studies and short-term experiments, mainly in rodents, have provided important insights into the role of gut microbiota in metabolic syndrome, additional approaches are needed to assess the nature of the complex interaction between host genetics, diet and the microbiota in the regulation of metabolism. Furthermore, a better and more detailed understanding of the interactions between the gut microbiota and peripheral and hepatic insulin resistance is needed. 

## Figures and Tables

**Table 1:** Characteristics of randomized clinical trials.

Reference	Country	Patients	Sample Size (Intervention/Control)	Probiotics	Probiotic Source	Dose (cfu/g)	Duration (Weeks)	Results
Andreasen et al. 2010 [114]	Denmark	T2D	21/24	*Lactobacillus acidophilus* NCFM	capsules	10^10^	4	preserved insulin sensitivity
Ejtahed et al. 2012 [115]	Iran	T2D	30/30	*L. acidophilus* La5 and *Bifidumbacterium lactis* Bb12	300 g/d probiotic or conventional yogurt	7.23 × 10^6^ 6.04 × 10^6^	6	FBG and HbA1c ↓, insulin ↔
Asemi et al. 2013 [126]	Iran	T2D	27/27	*L. acidophilus*, *L. casei*, *L. rhamnosus*, *L. bulgaricus*, *B. breve*, *B. longum* and *Streptococcus thermophilus*	capsules	2 × 10^9^ 7 × 10^9^ 1.5 × 10^9^ 0.2 × 10^9^ 20 × 10^9^ 7 × 10^9^ 1.5 × 10^9^ + 100 mg fructo-oligosaccharides	8	prevented rise in FBG, HOMA IR ↑, trend in hs-CRP ↓, GSH ↑
Mazloom et al. 2013 [127]	Iran	T2D	16/18	*L. acidophilus*, *L. bulgaricus*, *L. bifidum* and *L. casei*	capsules		6	FBG, insulin, QUICKI, HOMA IR, MDA, IL-6 and lipid profile ↔
Ivey et al. 2014 [121]	Australia	OW	Yogurt 40/37 Milk 39/40	*L. acidophilus* La5, *B. animalis subsp lactis* Bb12	Probiotic yogurt (+) probiotic capsule control milk (+) probiotic capsule	3.0 × 10^9^	6	probiotic yogurt vs. control milk: HOMA IR ↑, FBG, insulin or Insulin or HbA1c ↔ probiotic capsules vs. placebo: FBG ↑, other parameters of glycemic control ↔
Mohamadshahi et al. 2014 [116]	Iran	T2D, OW	22/22	*B. animalis* subsp. lactis Bb12 *L. acidpophilus* La5	300 g/d yogurt or conventional yogurt	3.7 × 10^6^	8	HbA1c and TNF-α ↓, FBG, hs-CRP and IL-6 ↔
Ostadrahimi et al. 2015 [117]	Iran	T2D	30/30	*L. casei*, *L. acidophilus*, *B. lactis*	600 mL/d probiotic fermented milk (kefir) vs. conventionally fermented milk	15 × 10^6^/25 × 10^6^/8 × 10^6^	8	FBG, HbA1c ↓, lipid profile ↔
Simon et al. 2015 [122]	Germany	H, OW	11/ 10	*L. reuteri* SD5865	capsule	2 × 10^10^	8	GLP-1, GLP-2 release ↑, insulin and C-peptide secretion ↑, insulin sensitivity, ectopic fat content, inflammation ↔
Firouzi et al. 2017 [119]	Malaysia	T2D	48/53	*L. acidophilus*, *L. casei*, *L. lactis*, *B. bifidum*, *B. longum* and *B. infantis*	powder	3 × 10^10^	12	HbA1c and insulin ↓, HOMA IR ↓, hs-CRP, lipid profile and blood pressure ↔
Mobini et al. 2017 [123]	Sweden	T2D	LD 15/HD 14/C 15	*L. reuteri* DSM 17938	powder	10^8^/10^10^	12 s	HbA1c, blood pressure, heart rate, lipid profile, liver fat, liver enzymes, leptin, adiponectin ↔
Tonucci et al. 2017 [118]	Brazil	T2D	25/25	*L. acidophilus* La5 and *B. animalis subsp. lactis* BB12	120 g/d probiotic goat milk vs. conventionally fermented goat milk	10^9^/10^9^	6	Fructosamin levels, TNF-α, Resistin ↓ trend in HbA1c ↓,

T2D, type 2 diabetes; FBG, fasting blood glucose; HbA1c, glycated hemoglobin, hs- CRP, high-sensitivity C-reactive Protein; GSH, glutathione; MDA, malondialdehyde; Il, interleukin; HOMA-IR, homeostatic model assessment; QUICKI, quantitative insulin sensitivity check index; GLP, glucagon-like peptide.
